# Anti-fibrotic potential of erythropoietin signaling on bone marrow derived fibrotic cell

**DOI:** 10.1186/s12882-021-02411-0

**Published:** 2021-05-31

**Authors:** Yasunori Iwata, Norihiko Sakai, Yuki Nakajima, Megumi Oshima, Shiori Nakagawa-Yoneda, Hisayuki Ogura, Koichi Sato, Taichiro Minami, Shinji Kitajima, Tadashi Toyama, Yuta Yamamura, Taro Miyagawa, Akinori Hara, Miho Shimizu, Kengo Furuichi, Takashi Wada

**Affiliations:** 1 Division of Infection Control, Department of Nephrology and Laboratory Medicine, Kanazawa University, 13-1 Takara-machi, 920-8641 Kanazawa , Japan; 2 Department of Nephrology and Laboratory Medicine, Kanazawa University, Kanazawa, Japan; 3 Division of Blood Purification, Kanazawa University Hospital, Ishikawa Kanazawa, Japan; 4 Division of Nephrology, Kanazawa Medical University School of Medicine, Ishikawa, Japan

**Keywords:** chronic kidney disease, fibricyte, erythropoietin, EPO receptor

## Abstract

**Introduction:**

The number of patients with end stage kidney disease (ESKD) are increasing world-side. While interstitial fibrosis (IF) is a common step for the progression to ESKD, therapeutic options for IF is still limited in clinical settings. We have reported that bone marrow-derived fibrotic cell, fibrocyte, is involved in the pathogenesis of kidney fibrosis. Also recent studies revealed that erythropoietin has protective effect on kidney diseases. However, it is unknown whether erythropoietin (EPO) inhibits fibrosis in progressive kidney injury. Therefore, we explored the impacts of EPO on kidney fibrosis with focusing on fibrocyte.

**Method:**

Fibrocyte was differentiated from peripheral mononuclear cells of healthy donor. Fibrocyte was stimulated with transforming growth factor beta (TGF)*-*β with/without EPO treatment. Moreover, the therapeutic effect of EPO was evaluated in murine unilateral ureteral obstruction (UUO) model.

**Result:**

TGF-β stimulation increased the expression of *COL1* mRNA in fibrocyte. EPO signal reduced the expression of *COL1* mRNA in dose dependent manner. EPO reduced mitochondrial oxidative stress and ameliorated mitochondrial membrane depolarization induced by TGF-β stimulation. Moreover, EPO reduced the mRNA expression of mitochondria related molecules, *TRAF6*, in fibrocyte. In addition, the count of CD45+/αSMA + double-positive fibrocyte was decreased in the EPO-administered UUO kidneys.

**Conclusion:**

EPO signals function to prevent kidney fibrosis, particularly in fibrocyte. Regulating the renal accumulation of fibrocyte is a part of the anti-fibrotic functions of EPO.

**Supplementary Information:**

The online version contains supplementary material available at 10.1186/s12882-021-02411-0.

## Introduction

Patients with end stage renal disease (ESRD) are ever-increasing in Japan, reaching over 340,000 people in total (http://www.jsdt.or.jp/overview_confirm.html). The overall medical expenditures for kidney diseases have amounted to approximately 1.5 trillion yen (http://www.seikatsusyukanbyo.com/statistics/2016/009223.php). At present, it is critically challenging to improve chronic kidney disease (CKD) prognosis in terms of medicine, socioeconomics, and medical economics.

Studies have revealed that chronic inflammation and ensuing fibrosis are common steps related to the etiological mechanism of progression to ESKD, regardless of the type of disease. Although various factors are involved in the process of fibrosis, we have focused on bone marrow-derived fibrotic cells and reported their significance [1, 2]. In kidney fibrosis models using mouse unilateral ureteral obstruction (UUO) and human CKD, bone marrow-derived fibrotic cells, fibrocyte, are associated with exacerbation of fibrosis and progression to CKD.

Recombinant human erythropoietin is used to treat anemia in CKD patients. Nevertheless, there are cases of low response and resistance to erythropoietin (EPO). Previously, we reported antibodies inhibiting the EPO receptor (EPOR) (anti-EPOR antibodies). Subsequently, we identified anti-EPO and anti-EPOR antibodies in the blood of patients with autoimmune diseases, such as CKD accompanied by anemia and systemic lupus erythematosus [3, 4]. Recently, EPOR expression was detected in non-bone marrow/erythroblastic systems, including the kidneys and cardiovascular system; these studies revealed that EPO signals function to protect cells and organs [5, 6]. Moreover, we showed that anti-EPOR antibodies are responsible for the pathological activity of lupus nephritis in systemic lupus erythematosus [7]. Furthermore, we demonstrated that EPO signals exhibit anti-inflammatory effects in cultured intrinsic renal cells [8]. These results indicate that EPO signals function to protect organs in kidney diseases and suggest that EPO could be a novel therapeutic target and/or biomarker. However, it is unknown whether EPO inhibits fibrosis in progressive kidney diseases. Therefore, we explored the impacts of EPO on kidney fibrosis with focus on fibrocyte.

## Materials and methods

### Cell culture

Human CD45+/COL1 + double-positive cells were obtained via the induced differentiation of peripheral mononuclear cells of three healthy women aged 22–27 years. Briefly, the mononuclear fraction was obtained by density gradient centrifugation techniques. The resultant cell resuspended in Dulbecco’s modified Eagle medium (DMEM) (Life Technologies) containing 1 % penicillin/streptomycin (Sigma-Aldrich) and 20 % fetal bovine serum (FBS) (Sigma-Aldrich). After attaining confluency, the cells were reinoculated into a 24-well plate at a ratio of 1 × 10^5^ cells/well and starved for 12 h in DMEM containing 1 % FBS. Finally, 1, 3, 5, 8, and 10 ng/mL human recombinant TGF-β (R&D systems) and 5, 25, 100, and 500 ng/mL human recombinant EPO (Merck Millipore) were added and the plate was left for 24 h.

### Unilateral ureteral obstruction (UUO) mouse model

 Animal experiments were conducted based on the Animal Experimentation Guidelines of Kanazawa University. Eight-week-old male C57BL/6 were purchased from Charles River Laboratories Japan (Tokyo, Japan). After anesthesia administration, the left ureter of each mouse was ligated using a suture thread through a low midline abdominal incision. Either 2.5 µg/kg mouse recombinant EPO (R&D systems) or 0.1 % Alb-added PBS (control solution) was subcutaneously administered at the day of ureter ligation. Then, either 2.5 µg/kg mouse recombinant EPO or 0.1 % Alb-added PBS was administered on alternate days. Three mice were allocated in each group. The mice were sacrificed at 7 days after ureter ligation. Both kidneys were harvested from the sacrificed mice and renal capsules were removed and stored at − 80 °C. Unobstructed contralateral kidneys were analyzed as controls.

### RNA extraction and cDNA preparation

Total RNA was extracted from the cultured cells using either the High Pure RNA Isolation Kit (Roche) or High Pure RNA Tissue Kit (Roche) according to the manufacturers’ instructions. The extracted total RNA (100 ng) was reverse-transcribed to cDNA. Reverse transcription (RT) was performed using the High Capacity cDNA Reverse Transcription Kits (Life Technologies) according to the manufacturer’s instructions.

### Quantitative polymerase chain reaction (qPCR)

In each PCR tube (Life Technologies), cDNA (1 µL/tube), iQ™ SYBR Green Supermix (5 µL), forward primer (0.2 µL), reverse primer (0.2 µL), and dH_2_O (3.6 µL) were added. qPCR was performed using the 7900HT Sequence Detection System (Life Technologies). The results were calibrated using glyceraldehyde-3-phosphate dehydrogenase (GAPDH) and analyzed by the Ct^−ΔΔ^ method.

### Primers

The primers used for qPCR were designed using the Primer Express software (Life Technologies). The primer sequences used to identify the respective genes are listed in Supplementary Table 1. The primers for GAPDH were purchased from Life Technologies.

### Evaluation of mitochondrial reactive oxygen species (ROS) production

Mitochondrial ROS production in culture cells stimulated with transforming growth factor (TGF)*-*β and EPO was evaluated using the MitoSOX™ Red Mitochondrial Superoxide Indicator (Thermo Fisher Scientific) according to the manufacturer’s instructions. The cells were observed under a fluorescence microscope (Biorevo BZ-9000, Keyence). Additionally, flow cytometry was performed to quantify the amount of dyed cells (FACS Calibur, BD Biosciences). Finally, the results were analyzed using the FlowJo software (Tree Star).

### Evaluation of mitochondrial membrane potential (MMP)

MMP in culture cells stimulated with TGF-β and EPO was evaluated using the MitoProbe™ JC-1 Assay Kit (Thermo Fisher Scientific) according to the manufacturer’s instructions. The cells were observed under a fluorescence microscope (Biorevo BZ-9000, Keyence).

### Quantification of hydroxyproline content in mouse kidneys

Kidney fibrosis in the UUO mice model was evaluated based on hydroxyproline quantification. The standard was measured along with the prepared samples for OD values at 550 nm to draw a standard curve.

### Fluorescent immunostaining of mouse kidney tissues

To evaluate the infiltration/accumulation of bone marrow-derived fibrotic cells in the kidneys of the UUO mice model, double staining of CD45 and αSMA was performed using of formalin-fixed paraffin-embedded kidney tissues. For αSMA staining, the M.O.M. Immunodetection Kit (Vector Laboratories) was used as per the manufacturer’s instructions. Anti-CD45 (R&D systems) was used, followed by biotinylated anti-Rat IgG (Vector Laboratories) and streptavidin 488 (Vector Laboratories). After mounting with Vectashield with DAPI (Vector Laboratories), the sample was observed under a fluorescence microscope (Biorevo BZ-9000, Keyence).

### Study approval

 All of the animals were maintained and used according to Kanazawa University guidelines, and the experiments were conducted under study approval No. AP-163,724 from the ethics committee of Kanazawa University. All procedures were carried in accordance with the ARRIVE guidelines. Human samples were collected with the approval of the ethics committee of Kanazawa University (IRB approval No. 285). All the experiments were conducted with the Declaration of Helsinki. All participants provided written informed consent and were informed about their right to withdraw from the study at any time.

### Statistical analysis

The results were statistically analyzed using the unpaired Student’s *t*-test. The values are represented as mean ± SE, with p < 0.05 regarded as statistically significant.

## Results

### Effects of TGF-β stimulus and EPO signals on collagen production and EPO receptor expression in fibrocyte

**Fig. 1:**
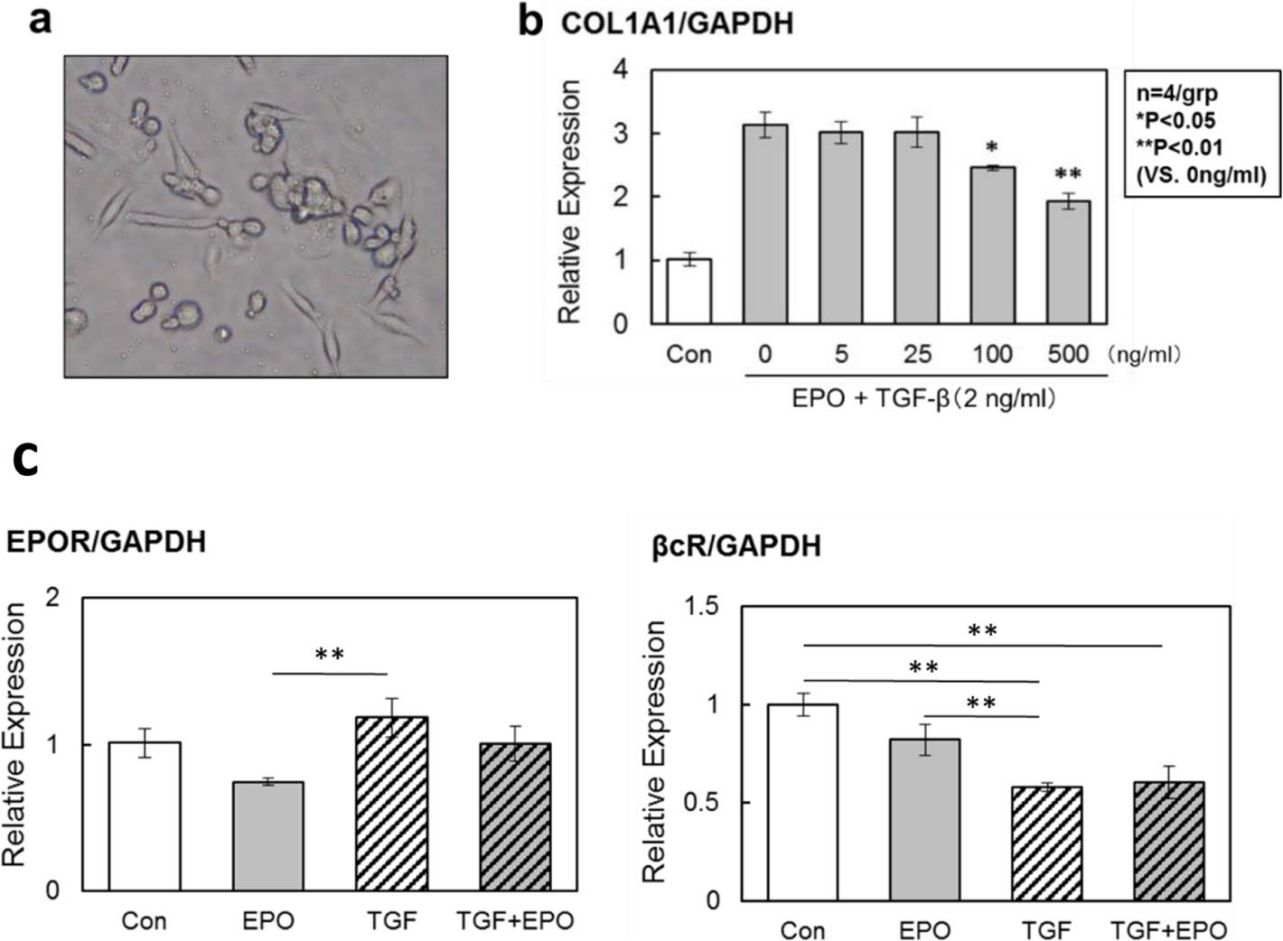
The mRNA expression levels of COL1 and EPO receptors were affected by TGF-β stimulation and EPO treatment in fibrocyte. Bone marrow derived fibrotic cells were isolated from PBMC of healthy control (**a**). TGF-β stimulation increased the expression of COL1 mRNA. EPO signal reduced the expression of COL1 mRNA in dose dependent manner (**b**). TGF-β stimulation induced the expression of EPOR (**c**), however it reduced the expression of βCR (**d**) in bone marrow derived fibrotic cells. EPO signaling did not change the expression levels of these receptors. Figures are representative of four independent experiments

Fibrocytes were isolated from the peripheral blood of healthy human individuals and cultivated. On day 5 of the cultivation, the cells extended cell processes, a morphology characteristic of fibrocytes (Fig. 1a). TGF-β stimulation approximately tripled the mRNA expression of CoL1. This TGF-β-accelerated expression did not significantly differ following the addition of 5 and 25 ng/ml EPO. However, when EPO was added at 100 and 500 ng/ml, the expression was suppressed in a concentration-dependent manner (Fig. 1b). The above results suggest shown that EPO signals suppress TGF-β-stimulated collagen production in fibrocytes.

There are two types of EPO receptors: one in which EPOR forms a homodimer and one in which βCR and EPOR form a heterotrimer [9, 10]. To examine the effects of TGF-β stimulus and EOP signals on the expression of these receptor genes, we performed qPCR to measure the gene expression levels of EPOR and βCR in fibrocytes. The mRNA expression level of EPOR decreased by EPO addition compared with the control. However, TGF-β stimulation did not lead to any changes. In contrast, the mRNA expression level of βCR decreased following TGF-β stimulation compared with the control; however, EPO addition did not result in any changes (Fig. 1c).

### Effects of TGF-β stimulus and EPO signals on mitochondrial oxidative stress and membrane potential

**Fig. 2:**
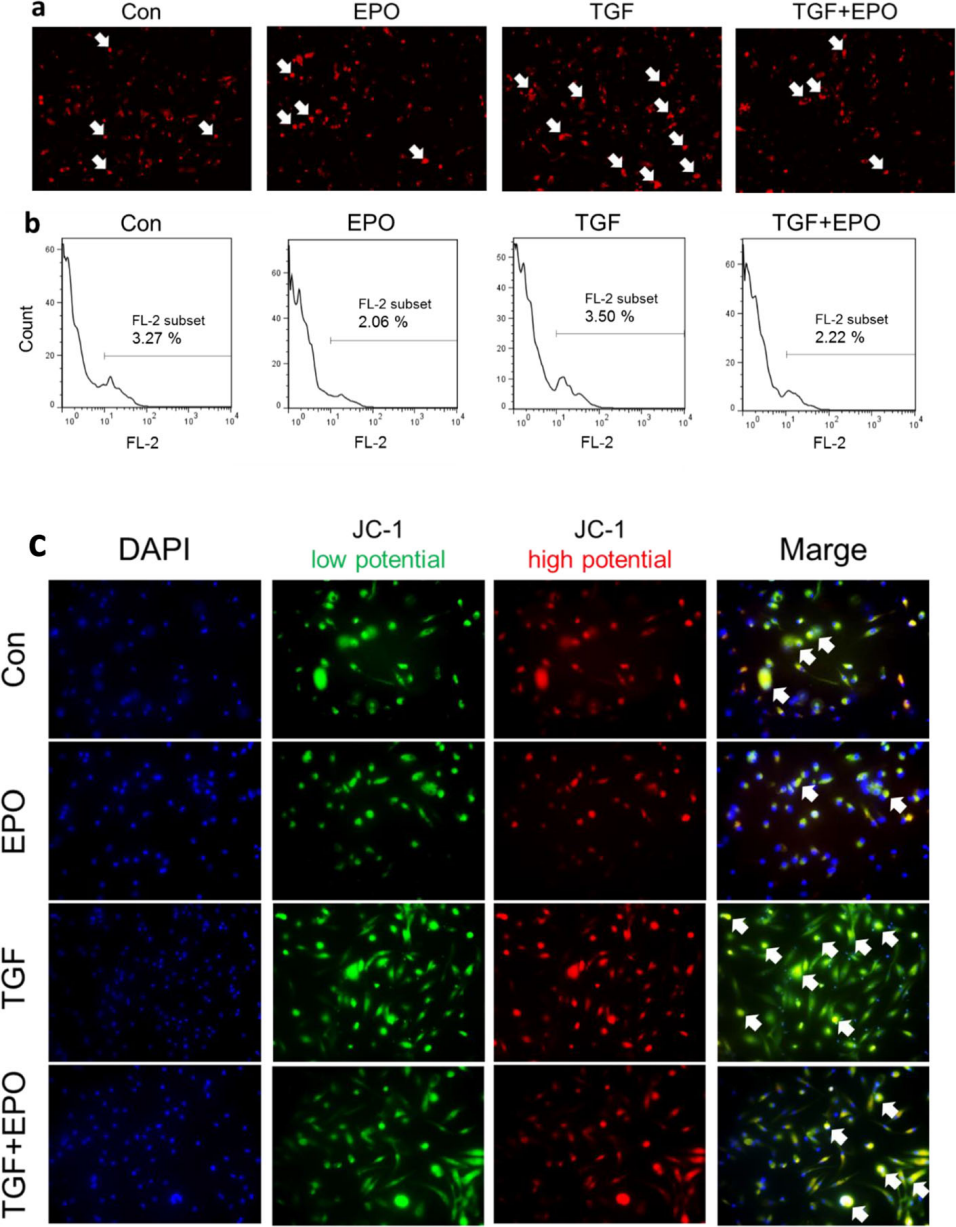
EPO reduced mitochondrial ROS production and attenuated mitochondrial membrane depolarization in TGF-β stimulated fibrocyte. Recent studies revealed the pathogenesis of mitochondria in organ fibrosis. We analyzed mitochondrial ROS production by mito-SOX reagent in bone marrow derived fibrotic cells. TGF-β stimulation increased mitochondrial ROS production as compared to no stimulated cells. The mitochondrial production of ROS was reduced by EPO signaling (**a**). Flow cytometry analysis also showed higher frequency of mito-SOX positive cells in TGF-β stimulated group. EPO treatment decreased the frequency of those cells (**b**). Mmembrane potential (MMP) were measured with JC-1 reagent. JC-1 red indicated mitochondria with high membrane potential, whereas JC-1 green indicated those with low membrane potential. TGF-β stimulation lowered MMP, that was attenuated by EPO signaling (**c**)

The involvement of mitochondrial pathology has been recently reported in models of various inflammatory diseases and fibrosis [11]. However, it is not clear how mitochondria are related to fibrosis in fibrocytes. Then, we investigated the effects of TGF-β stimulus and EPO signals on mitochondrial ROS production using MitoSox. During the observation, as indicated by a white arrow, the intensity of red fluorescence increased in the TGF-β-stimulated group compared with that in the control and EPO-administered groups. Conversely, the ROS production decreased in the TGF-β/EPO-added group compared with that in the TGF-β-stimulated group (Fig. 2a). In flow cytometric analysis, the proportion of MitoSox-positive cells slightly increased in the TGF-β-stimulated group compared with that in the control group. Meanwhile, the proportion decreased in the EPO-administered group compared with that in the control group. Therefore, EPO addition suppressed mitochondrial ROS production (Fig. 2b). Furthermore, we investigated the effects of TGF-β stimulus and EPO signals on MMP using JC-1. As indicated by a white arrow, in TGF-β-stimulated fibrocytes, the proportion of green fluorescence intensity relative to that of red fluorescence intensity increased compared with that in the control group, suggesting the occurrence of mitochondrial membrane depolarization. In contrast, in the TGF-β/EPO-added group, MMP nearly returned to normal. In the EPO-administered group, the proportion of green fluorescence relative to that of red fluorescence slightly decreased compared with that in the control group (Fig. 2c).

### Effects of EPO on the mRNA expression of mitochondria related molecules

**Fig. 3:**
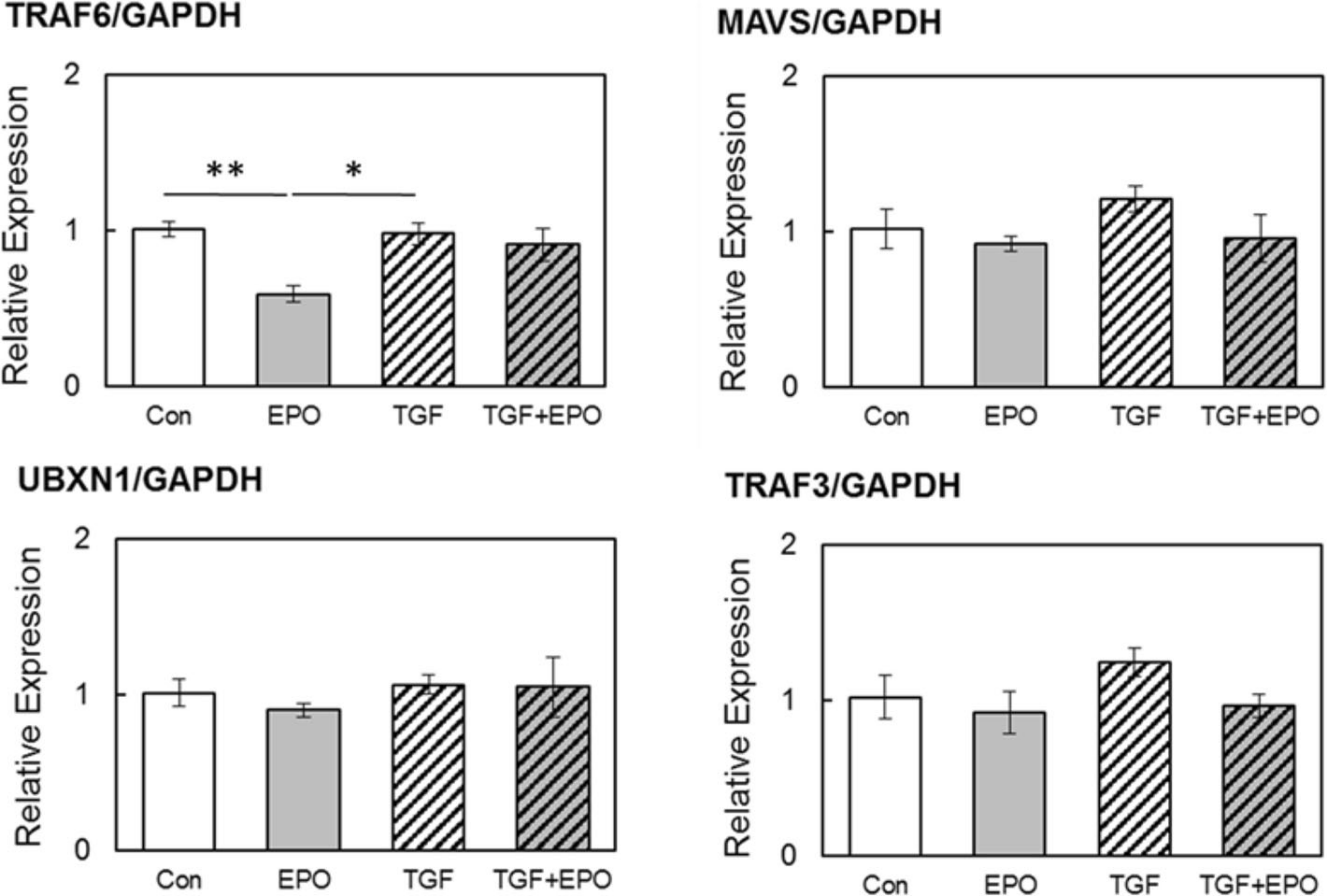
EPO signal tended to reduce the mRNA expression of tumor necrosis factor receptor-associated factor (TRAF)6 in fibrocyte. Next, we explored the mitochondria related signaling in TGF-β stimulated fibrocyte. EPO signal tended to reduce the mRNA expression of TRAF6, which is downstream signaling of Mitochondrial antiviral signaling (MAVS) signal. However, TGF-β stimulation did not attribute to the protein expression of other molecules

To determine whether EPO changes the expression of mitochondria related molecules, we analyzed the gene expression levels of Mitochondrial antiviral signaling (MAVS), UBX domain-containing protein 1 (UBXN1), Tumor Necrosis Factor receptor-associated factor (TRAF)3, and TRAF6 via qPCR. The mRNA expression of TRAF6 decreased by approximately 41 % in the EPO-administered group compared with that in the control group. In contrast, the mRNA expression of TRAF6 was not significantly different in the TGF-β-stimulated group compared with that in the control group. Meanwhile, the mRNA expression levels of MAVS, UBXN1, and TRAF3 did not significantly differ compared with those in the control group in any of the EPO-added, TGF-β-stimulated, and TGF-β/EPO-added groups (Fig. 3).

### Involvement of EPO signals in a mouse model for kidney fibrosis

**Fig. 4:**
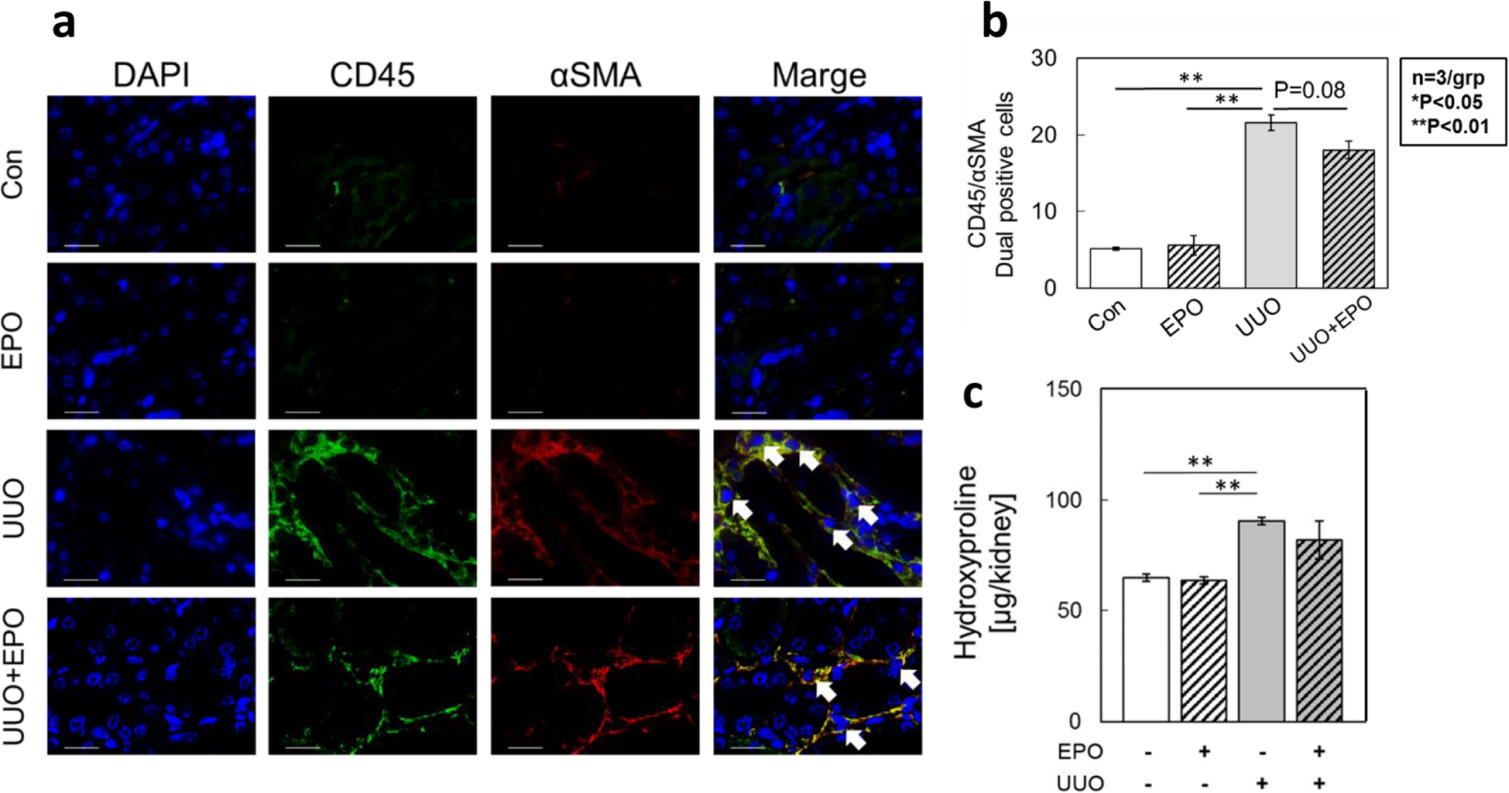
EPO signaling inhibited the accumulation of CD45+/αSMA + cells in mouse UUO model. The number of CD45+/αSMA + cells was analyzed by immunofluorescent staining. The dual positive cells for CD45+/αSMA + were accumulated in mouse UUO model. The administration of EPO inhibited the accumulation of CD45+/αSMA + cells (**a**, **b**). Nevertheless, compared with the PBS-administered group, hydroxyproline content did not differ in the UUO kidneys of C57BL/6 mice in the EPO-administered group (**c**). (Scale bars : 20 μm.)

Next, we explored the in vivo effects of EPO on kidney fibrosis using murine UUO model. The count of CD45+/αSMA + double-positive fibrocyte in the EPO-administered UUO kidneys decreased by approximately 16 % compared with that in the PBS-administered UUO kidneys (Fig. 4a, b). Nevertheless, compared with the PBS-administered group, hydroxyproline content did not differ in the UUO kidneys of C57BL/6 mice in the EPO-administered group (Fig. 4c).

## Discussion

The present study revealed that EPO signals reduced collagen production in human fibrocyte subjected to TGF-β simulation. Moreover, EPOR expression was observed in fibrocyte. EPO signals reduced intracellular mitochondrial ROS, thereby suppressing abnormal depolarization of MMP. Additionally, EPO signals reduced the accumulation of fibrocyte in the kidney tissue.

EPO is primarily produced in neural crest-derived fibrotic cells in the renal interstitium [12]. As CKD progresses, EPO production decreases, resulting in symptoms, such as nephrogenic anemia. A study recently reported that EPOR exists in various cells and functions to protect tissues against damage as well as prevents fibrosis [13]. The anti-EPOR antibody contributes toward the aggravation of nephrogenic anemia by mainly inhibiting EPO signals [3, 4]. Moreover, this antibody is correlated to the pathological activities of not only anemia but also lupus nephritis [7]. These findings suggest that EPO signals function to protect tissues against kidney damage.

In this study, TGF-β induced mRNA expression of *CoL1* was suppressed in a concentration-dependent manner by EPO addition in fibrocyte. The fact that EPO signals suppress collagen production suggest the presence of anti-fibrotic properties at the cellular level. However, in an EPO administration experiment for TGF-β-stimulated glial cells, a decrease in the expression of extracellular matrix protein was observed with only a 10th of the concentration of EPO that suppressed collagen production in our study [14]. This report corroborates the results obtained in our study that EPO suppresses the expression of fibrosis-related molecules. Nevertheless, further research is warranted to clarify how differences in cells affect EPO dynamics and signal transduction related to the suppression of collagen production.

In this study, EPOR expression was observed in fibrocyte. Other than fibrocyte, EPOR expression was reported in macrophages in previous studies [15, 16]. EPO signals augment the phagocytic ability of macrophages as well as the production of anti-inflammatory cytokines; therefore, EPO signals are considered to contribute toward immune regulatory phenotype of macrophages. The EPO receptor is divided into two types: one in which EPOR forms a homodimer and one in which EPOR and βCR form a heterotrimer. The organ protective functions of EPO signals have been reported to be attributed to signals mediated by EPOR/βCR heterotrimers [9, 10]. In an EPO administration experiment in a kidney fibrosis model, the count of fibrocyte decreased [17]. However, no studies focusing on EPOR and collagen production have been conducted. Therefore, the detection of EPOR and βCR in our study should constitute a new finding. Admittedly, this study only conducted gene expression analysis for βCR; therefore, further research is warranted to investigate how βCR protein expression and activity of each receptor are affected by TGF-β stimulation and EPO addition.

Recent studies revealed that EPO signaling protect mitochondria injury in cardiomyocytes [18] and pancreatic β−cells [19]. Moreover, mitochondrial fission and depolarization are related to collagen synthesis in fibroblasts [20]. Therefore, we evaluated mitochondrial ROS production and membrane potential in fibrocyte in association with EPO. In fibrocyte subjected to TGF-β stimulation, mitochondrial ROS production increased and the membrane potential was depolarized. These findings suggest that mitochondria are activated to produce ROS during collagen production in fibrocyte. In a previous study, mitochondrial ROS production in fibroblasts was reported to induce fibrosis [21]. A similar mechanism may come into play in fibrocyte. Moreover, the fact that EPO addition suppresses mitochondrial ROS production suggests that EPO signals are mediated by mitochondria and function to prevent fibrosis. In addition, an analysis was conducted using JC-1 to specifically visualize MMP. In this study, MMP depolarization caused by TGF-β stimulation was improved by EPO addition. These results suggest that mitochondrial activation is involved in TGF-β-stimulated collagen production in bone marrow-derived fibrotic cells and that this activation is regulated by EPO signals.

In the present study, we experimented on the assumption that mitochondria-related signals are involved in the anti-fibrotic functions of EPO. EPO addition decreased the mRNA and protein expression levels of TRAF6. In contrast, TGF-β stimulus did not significantly change the expression of mitochondria-related factors. No previous study has reported any relationship between TRAF6 and EPO signals; therefore, this should constitute a new finding. Meanwhile, TRAF6 has been revealed to be involved in several signal transduction pathways, including mitogen-activated protein kinase [22]. Considering with the reduced activation of mitochondria by EPO treatment, EPO signal may modulate mitochondrial functions. However, precise mechanisms still remain to be elucidated.

Additionally, to evaluate the in vivo anti-fibrotic effects of EPO signals, we administered EPO to the kidney fibrosis mouse model. In B6 mice, the count of fibrocyte significantly increased, which was slightly decreased after EPO administration. Combined with the aforementioned results of fibrotic suppression, the findings of the present study suggest that EPO signals are partly responsible for the kidney anti-fibrotic mechanism, thereby regulating the accumulation of fibrocyte in the kidneys. Nevertheless, hydroxyproline content did not decrease even in EPO treated mice. More details are need to be clarified in the relation between kidney fibrosis and EPO signal.

## Conclusions

Taken together, this study demonstrated that EPO signals function to prevent kidney fibrosis, particularly in fibrocyte. Moreover, regulating the renal accumulation of fibrocyte is a part of the anti-fibrotic functions of EPO. Further research is warranted to clarify the in vivo dynamics of EPO and determine how EPO signals affect the survival and proliferation of fibrocyte.

## Supplementary Information

**Additional file 1:**

## Data Availability

The data analyzed in the manuscript are available from the corresponding author on reasonable request.
